# Ensemble stacking mitigates biases in inference of synaptic connectivity

**DOI:** 10.1162/NETN_a_00032

**Published:** 2018-03-01

**Authors:** Brendan Chambers, Maayan Levy, Joseph B. Dechery, Jason N. MacLean

**Affiliations:** Committee on Computational Neuroscience, University of Chicago, Chicago, IL, USA; Department of Neurobiology, University of Chicago, Chicago, IL, USA

**Keywords:** Network analysis, Network motifs, Simulation and modeling, Synaptic connectivity, Information theory, Ensemble learning

## Abstract

A promising alternative to directly measuring the anatomical connections in a neuronal population is inferring the connections from the activity. We employ simulated spiking neuronal networks to compare and contrast commonly used inference methods that identify likely excitatory synaptic connections using statistical regularities in spike timing. We find that simple adjustments to standard algorithms improve inference accuracy: A signing procedure improves the power of unsigned mutual-information-based approaches and a correction that accounts for differences in mean and variance of background timing relationships, such as those expected to be induced by heterogeneous firing rates, increases the sensitivity of frequency-based methods. We also find that different inference methods reveal distinct subsets of the synaptic network and each method exhibits different biases in the accurate detection of reciprocity and local clustering. To correct for errors and biases specific to single inference algorithms, we combine methods into an ensemble. Ensemble predictions, generated as a linear combination of multiple inference algorithms, are more sensitive than the best individual measures alone, and are more faithful to ground-truth statistics of connectivity, mitigating biases specific to single inference methods. These weightings generalize across simulated datasets, emphasizing the potential for the broad utility of ensemble-based approaches.

## Author Summary

Mapping the routing of spikes through local circuitry is crucial for understanding neocortical computation. Under appropriate experimental conditions, these maps can be used to infer likely patterns of synaptic recruitment, linking activity to underlying anatomical connections. Such inferences help to reveal the synaptic implementation of population dynamics and computation. We compare a number of standard functional measures to infer underlying connectivity. We find that regularization impacts measures heterogeneously, and that individual algorithms have unique biases that impact their interpretation. These biases are nonoverlapping, and thus have the potential to mitigate one another. Combining individual algorithms into a single ensemble method results in a stronger inference algorithm than the best individual component measure. Ensemble-based inference can yield higher sensitivity to underlying connections and an improved estimate of the true statistics of synaptic recruitment.

## INTRODUCTION

Propagation of activity within neuronal networks is largely determined by underlying synaptic connectivity (Gerstein & Perkel, 1969; Kumar, Rotter, & Aertsen, 2010; Lindsey, Morris, Shannon, & Gerstein, 1997). This link has been demonstrated using recordings from pairs and small groups of neurons and has provided insights into plasticity processes (Kruskal, Li, & MacLean, 2013; Lalanne, Abrahamsson, & Sjöström, 2016), circuit structure (Ko et al., 2011; Perin, Berger, & Markram, 2011; Song, Sjöström, Reigl, Nelson, & Chklovskii, 2005), and noise correlations (Hofer et al., 2011). While methods such as paired patch clamp recordings or electron microscopy provide unambiguous indication of a synaptic connection, they are technically limited to the examination of a small number of connections with unknown functional relationships. Consequently, the statistics of circuit connectivity at the mesoscopic scale are difficult to conclude because of finite size effect errors (Vegue, Perin, & Roxin, 2017). In contrast, measures of dynamics, such as those generated by two-photon imaging of calcium fluorescence indicators (Sadovsky et al., 2011), allow up to 1,000 neurons to be recorded but require that synaptic connections be inferred using statistical dependencies in spike timing. Because neuronal spiking in neocortical networks requires synaptic input, the causal relationship between connectivity and activity can be exploited to infer network topology in direct relation to synaptic recruitment (Chambers & MacLean, 2015). In this framework, statistical dependencies in the spiking activity between pairs of neurons within a population are summarized as a weighted directed graph, and this weight matrix is informative about the likelihood of synaptic connections as well as their functional relationship. Only those synapses directly contributing to spike-time dependencies can be captured and summarized by these weight matrices (Chambers & MacLean, 2015). While this lessens the number of synaptic connections that can be inferred, this subset of connections (which we have referred to as the “recruitment” network) has particular importance for the propagation of spiking and is a desirable target for inference (Chambers & MacLean, 2016). Study of the link between structural and functional connectivity has the promise to reveal mechanistic insights as to how information flow is directed across networks, and the number of studies employing inference algorithms has grown rapidly. In this paper we compare and contrast the performance of a number of common inference methods, identify biases specific to individual inference methods, and then combine them in an ensemble to mitigate these biases and consequently improve inference of synaptic connectivity within large networks of neurons.

The importance of bridging function and structure is highlighted by the increasing diversity of methods for predicting synaptic connectivity from spiking activity. These efforts encompass methods based on counting lagged firing events (Pajevic & Plenz, 2012), lagged correlation (Sadovsky & MacLean, 2013), mutual information (Endo, Santos, Simpson, Maciel, & Newland, 2015), and transfer entropy (Ito et al., 2011; Stetter, Battaglia, Soriano, & Geisel, 2012) sometimes also referred to as conditional mutual information (Zhang, Zhao, Hao, Zhao, & Chen, 2015), as well as other approaches. Crucial to the performance of these methods is the time resolution of the spike trains, or the bin size in the binning procedure (Chambers & MacLean, 2015) over relevant timescales relating spiking to synaptic connectivity and integration. Consequently, we consider a range of bin widths in this work. The majority of these metrics are formulated in terms of correlations between consecutive time-bins, which implies causality. However, as the number of neurons densely recorded with imaging increases, acquisition time generally increases as well, and the relevant correlations may shift from consecutive time-bins towards simultaneous time-bins. We thus propose variations of information theoretic measures that account for simultaneous and joint time-bin correlations to reflect common experimental constraints.

Because each of the inference algorithms differentially quantifies statistical features of population dynamics, it is possible that they identify nonidentical sets of connections. Therefore, it is opportune to turn to progress in machine learning and bioinformatics, which have shown that combining approaches has the potential to pool over strengths and neutralize weaknesses of their constituent algorithms (Marbach et al., 2012). Collectively, these strategies are known as ensemble methods. An early formal description of an ensemble method was introduced to address the difficulty of optimization under conditions of multiple local minima in neural networks (Hansen & Salamon, 1990). Ensemble learning has been shown to occur in both the frequentist and Bayesian frameworks of machine learning exemplified respectively by boosting (Freund & Schapire, 1995; Schapire, 1990) and Bayesian optimal classifiers (Dietterich, 2000). In diverse settings, combinations of algorithms can be stacked together to yield an aggregate that outperforms its components (Fast & Jensen, 2008). However, it is unclear whether an ensemble approach can be applied to the problem of synaptic inference. We investigate this issue using simulated naturalistic spiking networks, where true underlying connectivity is known in full. Computational models are well suited to investigating synaptic topology, because they present transparent access to synaptic connectivity and spike timing. Prior work has demonstrated that random synaptic networks present the greatest challenges for inferring synaptic connections from activity (Kobayashi & Kitano, 2013). Therefore, although synaptic networks in the neocortex are known to be nonrandom, random models are useful for benchmarking success rates in synaptic inference applications. It is also important to consider naturalistic regimes of population activity, because network state can determine the success or failure when inferring synaptic connections (Stetter et al., 2012). To compare diverse inference methods and judge whether they may complement one another, we designed network simulations to reflect naturalistic dynamics and reflect experimental constraints. We show that an ensemble approach reveals a more extensive subset of the synaptic network, and one that is more faithful to the true statistics of the synaptic recruitment network measured in our simulations. A host of weighted combinations improve over the best individual measures, and these weighting schemes are transferrable from one simulated dataset to another.

## METHODS

### Network Simulations

**Figure 1.**
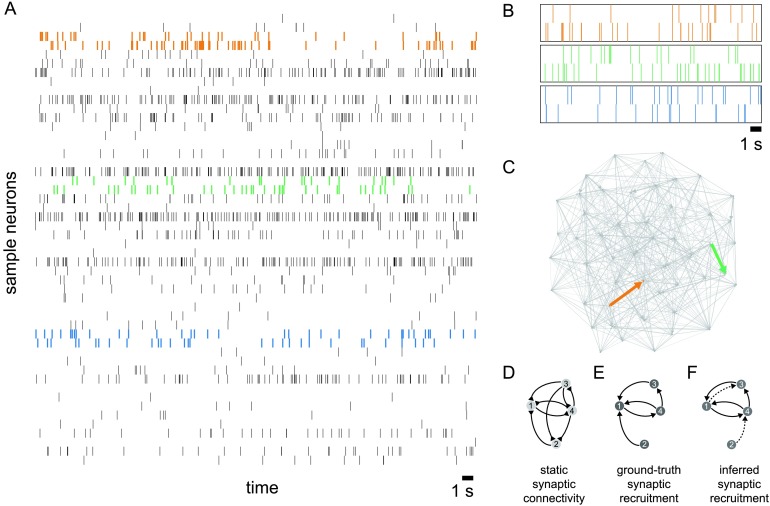
Inferring synaptic connectivity from pairwise spike timing. (A) Population spike raster for 50 random excitatory model neurons during 40 s simulated recording. Three representative pairs matched for firing rates are shown in color: strongly connected (orange), weakly connected (green), and unconnected (blue). Spikes were binarized at 20 ms time-bins. (B) The same example pairs as in panel A during another 20 s of simulated recording. (C) Ground-truth synaptic connectivity for excitatory neurons shown in panel A. Edge width indicates weight. Arrows mark the strongly connected pair (orange) and weakly connected pair (green). Width was enhanced for visibility purposes. (D) Schematic of a synaptic network among four active neurons. (E) Synaptic recruitment is defined as lagged firing between pre- and post-synaptic pairs. Under the conditions of a given input, network state, and recording duration, not every synaptic connection recruits its post-synaptic partner to generate an action potential. (F) Inferred synaptic connectivity (solid lines) mirrors the recruitment network, mapping propagating activity. Errors occur when inference algorithms fail to detect sites of synaptic recruitment (e.g., missing edge from neuron 2 to neuron 1), or assign putative connectivity (dashed lines) where there is none in truth.

In order to obtain a ground truth for network connectivity, we constructed six networks of conductance-based leaky-integrate-and-fire (LIF) neurons, similar to previous work (Chambers & MacLean, 2015, 2016). Each simulated dataset was defined as a synaptic connectivity matrix (Figure 1C) with 1,000 excitatory units and 200 inhibitory units, constructed probabilistically according to a stochastic blocked model with naturalistic connection probabilities. The excitatory subnetwork taken in isolation formed an Erdos-Renyi graph (*p* = 0.2). Specifically, we set *P*_*ee*_ = 0.2, *P*_*ei*_ = 0.35, *P*_*ie*_ = 0.25, and *P*_*ii*_ = 0.3, with *e* denoting excitatory units, *i* denoting inhibitory neurons, and the first and second letters in the subscript standing for the pre- and post-synaptic neuron, respectively.

Each neuron’s membrane potential was governed by the following:

$$\tau_{m}\frac{dV}{dt}=g_{e}(E_{e}-V)+g_{i}(E_{i}-V)+g_{l}(E_{l}-V)+g_{tonic}(E_{tonic}-V),$$ (1)

$$\tau_{e}\frac{dg_{e}}{dt}=-g_{e},$$ (2)

$$\tau_{i}\frac{dg_{i}}{dt}=-g_{i}.$$ (3)

A spike occurred every time the membrane potential crossed a threshold, set at −48 mV. Post spike, membrane potential was then reset to −70 mV, and a 1-ms refractory period imposed. Conductances and equilibrium potentials were defined for leak (*l*), excitatory synapses (*e*), inhibitory synapses (*i*), and a tonic input serving to stabilize spiking (*tonic*) (Table 1).

**Table 1.** Parameters of the spiking network model

Parameter	Value	Parameter	Value
*E*_i_	−90 mV	*g*_l_	0.2 mS
*E*_e_	0 mV	*g*_tonic_	0.2 mS
*E*_l_	−65 mV	*τ*_m_	20 mS
*E*_tonic_	0 mV	*τ*_e_	10 mS
		*τ*_i_	5 mS

Synaptic weights were randomly sampled from a lognormal distribution with location and scale parameters *μ* = −0.64 and *σ* = 0.51. The resulting weights distribution had a mean of 0.6 and variance of 0.11, relative to the scale of the leak conductance. Since an important subset of inhibitory projections onto excitatory cells tend to synapse on the soma and proximal dendrites (Markram et al., 2004) and are thus more potent, we enhanced *I* to *E* weights by 50%. We started each simulation by initializing membrane potentials to values drawn randomly from a normal distribution with a mean of −65 mV and a standard deviation of 5 mV. A pool of 50 Poisson neurons was used as input to the network. Poisson neurons spiked at 15 Hz and were independently connected to excitatory units with P = 0.1 and 0.6 synaptic weight in the units of the leak conductance. The network was driven with the input pool for 50 ms and activity was allowed to continue for 100 ms, after which the simulation was terminated. This procedure was repeated over 100 trials with 10 different inputs. All simulations were carried out using the Brain Simulator (Goodman & Brette, 2009), with Euler’s method for integration and time steps of 1 ms.

### Inference Measures

Spikes were binned in six time resolutions (1, 5, 10, 20, 40, and 80 ms) into time frames containing binary values, resulting in 150,000, 30,000, 15,000, 7,500, 3,750, and 1,875 bins, respectively. We employed seven pairwise measures of connectivity between neurons: lag count, abbreviated as count; lag correlation, abbreviated correlation; consecutive mutual information (cMI); simultaneous MI (sMI); confluent MI (conMI); first-order transfer entropy (TE [*k* = 1]); and second-order transfer entropy (TE [*k* = 2]). We thus consider a wide array of inference algorithms ranging in sophistication.

For each pair of neurons *i*,*j* we defined a binary variable $c_{ij}^{kl}(t)$, which evaluates to 1 if *i*(*t*) = *k* and *j*(*t* + 1) = *l*. For example,

$$\begin{array}{c} c_{ij}^{11}(t)=\left\{\begin{array}{cc} 1\;i(t)=1 & and\;j(t+1)=1\\ 0 & otherwise \end{array}\right.. \end{array}$$ (4)

Lag count was then calculated as

$$count_{ij}=\overset{T-1}{\underset{t=1}{\sum }}c_{ij}^{11}(t),$$ (5)

where *T* is the number of time-bins.

Lag correlation between two spike trains was calculated using the phi coefficient:

$$correlation_{ij}=\frac{\left[\overset{T-1}{\underset{t=1}{\sum }}c_{ij}^{11}(t)\cdot \overset{T-1}{\underset{t=1}{\sum }}c_{ij}^{00}(t)\right]-\left[\overset{T-1}{\underset{t=1}{\sum }}c_{ij}^{10}(t)\cdot \overset{T-1}{\underset{t=1}{\sum }}c_{ij}^{01}(t)\right]}{\sqrt{2(T-1)}}.$$ (6)

We use three versions of mutual information; consecutive mutual information (cMI) between a pair of neurons *i*, *j* was calculated as

$$cMI_{ij}=\underset{i(t)\in \{0,1\}}{\sum }\underset{j(t+1)\in \{0,1\}}{\sum }p(i(t),j(t+1))\cdot \log_{2}\left[\frac{p(i(t),j(t+1))}{p(i(t))\cdot p(j(t+1))}\right].$$ (7)

However, we note that binning spikes into longer time-bins may result in the pre- and post-synaptic spikes being binned into the same bin. Thus, we also consider simultaneous mutual information (sMI),

$$sMI_{ij}=\underset{i(t)\in \{0,1\}}{\sum }\underset{j(t)\in \{0,1\}}{\sum }p(i(t),j(t))\cdot \log_{2}\left[\frac{p(i(t),j(t))}{p(i(t))\cdot p(j(t))}\right],$$ (8)

and confluent mutual information (conMI),

$$conMI_{ij}=\underset{i(t)\in \{0,1\}}{\sum }\underset{j(\hat{t})\in \{0,1\}}{\sum }p(i(t),j(\hat{t}))\cdot \log_{2}\left[\frac{p(i(t),j(\hat{t}))}{p(i(t))\cdot p(j(\hat{t}))}\right],$$ (9)

where $j(\hat{t})=\left\{\begin{array}{cc} 1\;j(t)=1 & OR\;j(t+1)=1\\ 0 & otherwise \end{array}\right.\;.$

As nonsymmetric information theoretical measures we calculated transfer entropies under first- and second-order Markov models (TE1 and TE2, respectively) between every pair *i*, *j* of neurons:

$$TE1_{ij}=\underset{j(t),j(t+1),i(t)\in \{0,1\}}{\sum }p(i(t),j(t+1),j(t))\cdot \log_{2}\left[\frac{p(j(t))\cdot p(i(t),j(t+1),j(t))}{p(i(t),j(t))\cdot p(j(t+1),j(t))}\right].$$ (10)

TE2 is similarly defined, with the information *i*(*t*) is providing about *j*(*t* + 1) conditioned not only on *j*(*t*) but also on *j*(*t* − 1).

### Measure Evaluation

In order to evaluate performance of individual measures and the combined ensemble, we calculated the recruitment network for each model. The recruitment network (Figures 1D–F) is the intersection between the connectivity matrix and those synapses that directly contribute to post-synaptic firing, since these are the only synapses that can be inferred using spikes (see schematic; Chambers & MacLean, 2016). We first defined the active network in a similar way to *count*_*ij*_ (Equation 5), but modified $c_{ij}^{kl}(t)$ so that both consecutive and simultaneous time-bins are considered:

$$\begin{array}{c} {\hat{c}}_{ij}^{11}(t)=\left\{\begin{array}{cc} 1\;i(t)=1\;and & \left[j(t)=1\;OR\;j(t+1)=1\right]\\ 0 & otherwise \end{array}\right., \end{array}$$ (11)

$$active_{ij}=\overset{T-1}{\underset{t=1}{\sum }}{ĉ}_{ij}^{11}(t).$$ (12)

The recruitment network was then computed as a binary matrix:

$$\begin{array}{c} recruitment_{ij}=\left\{\begin{array}{ccc} 1\;active_{ij}>0 & AND & adj_{ij}>0\\ 0 & \;otherwise \end{array}\right.\;, \end{array}$$ (13)

where *adj*_*ij*_ is the adjacency matrix used to run the simulation.

The percentage of connections retained in the recruitment network out of the static synaptic connectivity is described in Table 2. Previously we have reported that it is only possible to infer connections that are active and temporally proximal to an action potential in the post-synaptic neuron (Chambers & MacLean, 2015). To reflect this fact we used the recruitment network as ground truth, and defined performance of an algorithm as the number of inferred connections at 80% true positive rate. This true positive rate was chosen based on survival curve analysis on three representative measures at all time resolutions (Figure 5). This definition provides a realistic test of performance, as only those active connections that contribute to spiking in the post-synaptic neuron can be captured by an inference algorithm of any kind. A lower threshold and increased coverage under the same true positive rate means that as the refinement process progresses, inferred adjacency matrices become sparser because false alarms are being removed from those matrices.

**Table 2.** Percentage of connections retained in the recruitment network

	5 ms	10 ms	20 ms	40 ms	80 ms
Simulated dataset 1	34.95%	40.82%	47.97%	53.49%	60.89%
Simulated dataset 2	41.41%	46.42%	53.18%	57.75%	65.22%
Simulated dataset 3	39.81%	45.63%	52.17%	56.70%	63.56%
Simulated dataset 4	45.72%	50.85%	58.57%	64.58%	72.32%
Simulated dataset 5	43.29%	49.38%	56.08%	61.04%	68.09%
Simulated dataset 6	42.32%	47.58%	54.36%	59.56%	67.14%

Data are shown for six simulated datasets binned at five time resolutions.

### Ensemble Weights and Scores

We employed a simulated annealing strategy with cyclical dynamics to search over weighted linear combinations of individual measures. Before pooling, individual measures were normalized by their maximum value to provide a uniform scale. The final inputs into the simulated annealing algorithm were then computed as the following:

$$S{\left(m\right)}_{ij}=\sqrt{\left|\frac{norm\_residual{\left(m\right)}_{ij}}{\max(norm\_residual(m))}\right|}\cdot \text{sgn}(norm\_residual{\left(m\right)}_{ij}),$$ (14)

where *norm_residual*_*ij*_ is the final transformation of every measure, and defined in the results (Equation 20); sgn denotes the sign; and *m* stands for the measure used, so each measure had its own *S*_*ij*_.

The random search algorithm was used to train weights independently on each of six simulated networks using coverage at 80% accuracy as our objective function (Figure 7A). This objective function proved to be pockmarked with many local maxima, motivating the use of repeated increment and cooling in the annealing process to avoid local maximum traps (Kirkpatrick, Gelatt, & Vecchi, 1983). As step size decreased, if no further gains were achieved, the search algorithm would occasionally jump to the last step size that did yield improvements. If these larger jumps still failed to improve the performance, the jump size was further increased. Weights were learned across five repeats of training for each model and timescale (Figure 7A, 10 ms).

An ensemble score was then calculated for each pair of neurons as a linear weighted sum of *S*(*m*)_*ij*_ (Equation 14):

$$Ensemble_{ij}=\overset{M}{\underset{m=1}{\sum }}w_{m}\cdot S{\left(m\right)}_{ij},$$ (15)

where *w*_*m*_ are the weights of the measures found by the annealing algorithm, and *M* is the number of measures.

### Comparison Between Inference Measures

All comparisons were conducted after completing the regularization steps described in the Results section, that is, on the normalized-residual adjacencies. Since summary statistics for adjacency matrices are impacted by edge density, inferred adjacency matrices were thresholded to match sparseness before conducting any comparisons, isolating just the strongest relationships for each measure. To match sparseness, thresholding was performed at the 98th percentile, leaving the top 2% of entries for each measure. Weights exceeding the inclusion threshold were mapped to 1 and those failing to reach inclusion threshold were mapped to 0. This pruning procedure allowed us to more clearly identify biases inherent to individual algorithms, and was not used to evaluate performance. Similarity between measures was assessed by vectorizing adjacency matrices and comparing the Euclidean distances separating each pair of measures. This comparison was performed independently for each simulated dataset. Reciprocity was quantified as the probability a randomly selected nonzero edge from neuron *i* to neuron *j* was accompanied by a nonzero edge from *j* to *i*. Local clustering for neuron *k* was quantified as the counted number of connected triangles including *k* divided by the number of possible triangles including *k*. In this formulation, a triangle must be composed of neuron *k* plus two immediate neighbors of *k*, without constraints on directionality. Thus, local clustering quantifies neighbor-of-neighbor relationships in the immediate neighborhood around *k*. Reciprocity and local clustering were aggregated by taking the mean over all edges and neurons, respectively, in the simulated dataset.

## RESULTS

### Simulated Neuronal Networks

Randomly connected recurrent networks (Figure 1C) composed of 1,000 excitatory and 200 inhibitory LIF neurons showed persistent naturalistic activity after being driven by a sparse set of Poisson inputs for 50 ms. Across six randomly connected networks, 99 ± 0.05% (mean ± *SD*) of neurons within the excitatory pool spiked at least once, and displayed sparse asynchronous-irregular firing (Figures 1A and 1B). Firing rates were 1.66 ± 3.30 Hz (mean ± *SD*) and followed a lognormal distribution, and single units showed irregular spiking consistent with experimental measures in cortical neurons (Destexhe, Rudolph, & Paré, 2003; Softky & Koch, 1993), with inter-spike interval coefficient of variation 1.04 ± 0.20 (mean ± *SD*). The numbers of spikes used for inference in the six simulated datasets varied and depended on the size of the time-bin used (Table 3). Using these simulated networks, which produced naturalistic spiking activity, we examined whether spiking activity within the network can be used to reveal underlying synaptic connectivity across the population. We employed sparse recurrent networks with random connectivity, constructed probabilistically according to naturalistic parameters (see the Methods section). Under some circumstances, nonrandom connectivity (e.g., local clustering) can facilitate inference of connections (Kobayashi & Kitano, 2013), so random synaptic connectivity may provide a more appropriate benchmark for comparing inference methods, particularly in assessing their propensity towards false-positive errors. Because connectivity in neocortex is not random (Song et al., 2005), the results presented here can be interpreted as a proof of concept of what is achievable when applying these inference approaches to experimental data. While it remains unclear how broadly results generated using randomly connected networks generalize, we have found that specific motifs of higher order correlations found in random networks are also found in spiking data collected from neocortex (Chambers & MacLean, 2016). There have been a number of studies that have examined whether correlation can be informative of causal connections, and it remains unclear how to best utilize the information provided by different inference approaches to uncover synaptic connectivity. As can be seen in Figure 1B, synchronous or lagged events occur for both strongly and weakly connected neuron pairs, as well as by chance for unconnected neurons, rendering the task of interaction inference difficult. Here we introduce a series of refinements to traditional connectivity measures and demonstrate improvement in our ability to correctly identify monosynaptic excitatory connections using spiking across a recorded population of neurons.

**Table 3.** Number of spikes per trial used for inference

	5 ms	10 ms	20 ms	40 ms	80 ms
Simulated dataset 1	135.13 ± 239.70	134.89 ± 238.94	130.12 ± 224.07	118.02 ± 189.30	102.41 ± 148.06
Simulated dataset 2	233.75 ± 513.30	233.34 ± 510.44	225.82 ± 471.20	205.27 ± 384.84	178.00 ± 286.54
Simulated dataset 3	264.25 ± 602.24	263.35 ± 595.56	251.24 ± 536.08	222.39 ± 425.01	187.51 ± 311.65
Simulated dataset 4	212.17 ± 340.30	212.02 ± 339.90	208.32 ± 339.47	196.14 ± 295.79	177.62 ± 245.71
Simulated dataset 5	282.00 ± 557.41	281.34 ± 554.79	269.80 ± 514.41	241.43 ± 424.08	205.72 ± 320.39
Simulated dataset 6	221.79 ± 424.11	221.58 ± 423.26	216.26 ± 404.11	200.09 ± 348.86	176.78 ± 274.40

Data represent mean ± *SD* for six simulated networks binned at five time resolutions.

### Signed Information Theoretic Measures: Removing Negative Correlations

**Figure 2.**
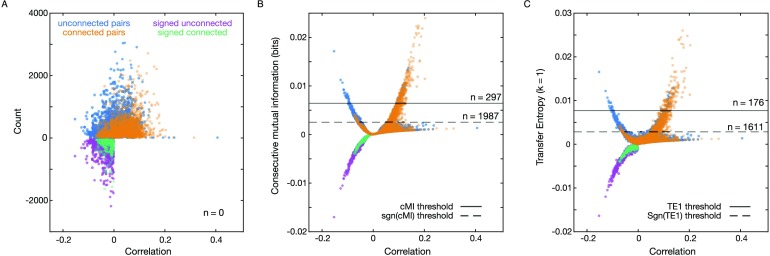
Leveraging anticorrelations to isolate excitatory connections. Data in this figure are from a randomly chosen representative simulated network binned at 20 ms, and subsampled according to density for display purposes. (A) Unconnected pairs often attained high lagged-count scores, but tended to exhibit negative-shifted lag correlations compared with connected pairs. Signing lag count scores on the basis of lag correlation thus improved performance, although signed lag count still fails to achieve sensitivity at the 80% accuracy threshold. (B) A subset of unconnected pairs exhibited high consecutive mutual information scores and strong negative lag correlations. Signing consecutive mutual information entries on the basis of lagged correlations dramatically improved sensitivity at the 80% accuracy threshold, increasing from 297 putative connections to 1,987 putative connections in the representative model dataset. (C) Transfer entropy is prone to the same errors, so that signing transfer entropy scores based on lag correlation extends coverage of putative connections from 176 to 1,611 directed pairs.

There are a number of approaches to inference. Some address the frequency of lagged or simultaneous spiking directly, such as the count method and the correlation method. For these measures, edge *e* = (*i*, *j*) is positive only if there is the possibility that neuron *i* recruited neuron *j* to fire—that is, only if *j* becomes active after *i* at least once (Figure 2A). In contrast, mutual information is related to lagged firing in a more abstract sense. As a practical consequence, the entries of *MI*_*ij*_ can be large given any type of interaction between the pre- and post-synaptic neurons. Indeed, we find that many pairs that were ranked highly under mutual information and transfer entropy had negative *correlation*_*ij*_ scores (Figures 2B and 2C). Unsurprisingly given the negative scores, the majority of those pairs were not monosynaptically connected, despite their high information theoretic scores. In order to account for interaction directionality consistent with causal synaptic interactions, we signed our mutual information and transfer entropy metrics on the basis of *correlation*_*ij*_:

$$signed{\left(X\right)}_{ij}=X_{ij}\cdot \text{sgn}(correlation_{ij}),$$ (16)

where *X* stands for the information theoretic measure considered (e.g., *signed*(*conMI*)_*ij*_ is the signed confluent mutual information between neurons *i* and *j*).

The signing procedure yielded gains in accuracy. These improvements are apparent when comparing the thresholds achieving 80% prediction accuracy for the raw versus signed metrics (Figures 2B and 2C). We confirmed the intuition that negative interactions can confound the detection of excitatory connections using information theoretic methods but can be accounted for. It remains an open question whether negative information theoretic scores are indicative of inhibitory connections. Inference of inhibitory connectivity is especially challenging, because of the ambiguity in distinguishing inhibition per se from the absence of excitatory drive. In this work, we consider only the positive entries of *signed*(*X*)_*ij*_, and denote those *pos*(*X*)_*ij*_.

### Removing Additional Spurious Correlations

Removing negative correlations allowed us to identify and correct for one source of false positives. Nevertheless, it remained clear that a significant overlap between our true positive signal and false-positive background is still present. We observed that false positives sometimes appeared to span source and target nodes with high weighted out- and in-degrees. Indeed, functional interactions are known to be heterogeneous with a heavy tail (Nigam et al., 2016; Sadovsky & MacLean, 2013; Shimono & Beggs, 2015), revealing indiscriminate patterns of spike-time coordination. Since experimental evidence indicated that synaptic connectivity is sparse, we reasoned that neurons with extremely high weighted degrees reflected coordination in activity not arising from monosynaptic connections alone, but rather coordinated population dynamics. For the purpose of identifying likely synaptic pairs, these are “background” spurious correlations. We estimated the magnitude of these background correlations, which depended on pre- and post-synaptic identity, and removed them from inferred weights.

Previous work employing mutual information to infer protein interactions had shown that removing spurious correlations by linear regression refined inference and preferentially identified residues known to interact physically (Little & Chen, 2009). Inspired by this work, we asked whether a similar correlation existed in the neuronal interactions of our networks. Noting that positive values of information theoretic scores have highly skewed distributions, we reexpressed the measures with the exception of count because of the discrete nature of the metric. Reexpression was performed by Tukey’s ladder of power (Tukey, 1977), which finds the exponent that minimizes a distribution skewness:

$$\begin{array}{c} a=\begin{array}{c} \text{arg min}\\ a \end{array}skewness(pos{\left(X\right)}^{a}). \end{array}$$ (17)

Each measure was reexpressed accordingly:

$$redist(X)=pos{\left(X\right)}^{a}.$$ (18)

Having reexpressed the scores, we next calculated the background signal for each pair of neurons. This was achieved by averaging the scores of the pre- and post-synaptic neurons when partnered with every other potential post- and pre-synaptic neuron in the network, respectively. This is equivalent to taking the mean across columns for the pre-synaptic neuron, and across rows for the post-synaptic neuron, excluding the partner neuron currently examined. We multiplied those mean scores for the pre- and post-synaptic neuron, denoted *background*_*ij*_:

$$background_{ij}=\langle redist(X_{i,1:N-\{j\}})\rangle \cdot \langle redist(X_{1:N-\{i\},j})\rangle ,$$ (19)

where 〈…〉 is the mean.

**Figure 3.**
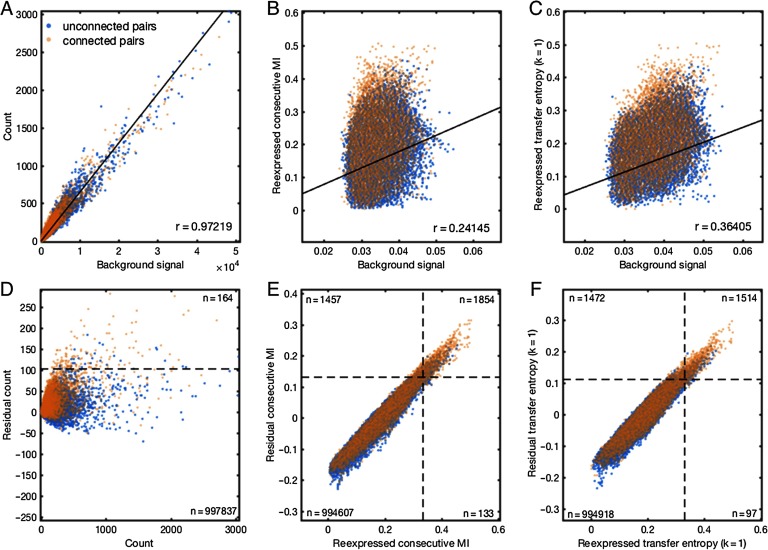
Removal of mean background timing relationships improves detection of synaptic pairs. Data in this figure are from a randomly chosen representative simulated network binned at 20 ms, and subsampled according to density for display purposes. (A) Linear regression revealed a strong background component in the count measure, reflecting a tendency for strong timing relationships to appear in tandem at select model neurons, encompassing both connected and unconnected pairs. (B) After reexpressing global weights for each measure to approximate normality, a weaker but significant background signal was revealed for the consecutive mutual information measure. (C) Background signal manifested somewhat more strongly for the transfer entropy (*k* = 1) measure. (D) Removal of mean neuron-wise background signal improved performance for the count measure, but unconnected pairs with high residual scores remained a serious obstacle to high performance. (E) For the consecutive mutual information measure, removal of background signal improved coverage at the 80% accuracy threshold from 1,987 (two right quadrants) to 3,311 (two top quadrants) putative pairs. (F) For the transfer entropy (*k* = 1) measure, removal of background improved coverage at the 80% accuracy threshold from 1,611 to 2,986 putative pairs.

A high average score, while potentially indicative of wide-reaching polysynaptic influence across the network, is unlikely to reveal monosynaptic connections. Indeed, *background*_*ij*_ was found to be highly correlated with *count*_*ij*_ (Figure 3A), and only to a lesser extent with information theoretic measures, although correlations were still significant (Figures 3B and 3C).

In order to remove the influence of this background signal, we calculated the residual of each measure, *residual(X)*_*ij*_, over *background(X)*_*ij*_ by linear regression. We found improved coverage with the residual scores over the reexpressed scores, with up to eightfold increase (93% increase on average) in connections uncovered with information theoretic measures corrected in this manner (Figures 3D and 3F).

### Scaling Residuals to Account for Heteroskedasticity

**Figure 4.**
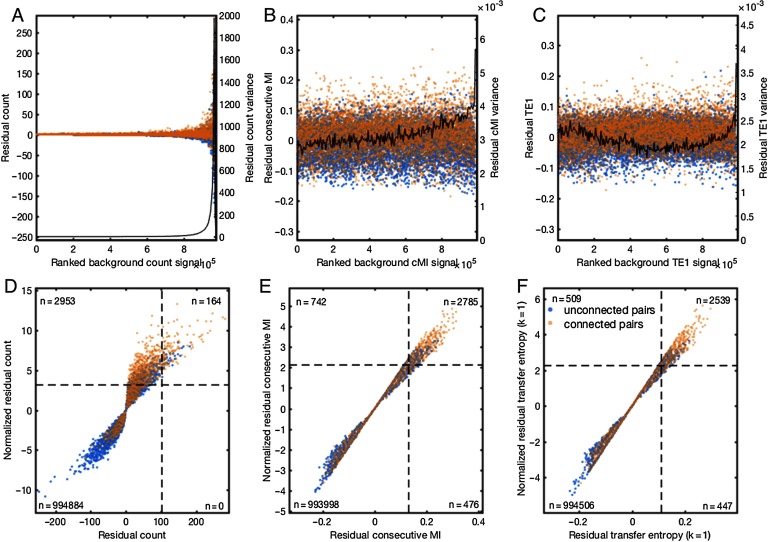
Rescaling neuron-wise residual score variance. Data in this figure are from a randomly chosen representative simulated network binned at 20 ms, and subsampled according to density for display purposes. (A) The count measure exhibited strong scaling of residual variance with mean background signal, negatively impacting performance after thresholding. (B) Consecutive mutual information exhibited modest heteroskedasticity. (C) Residual variance for transfer entropy (*k* = 1) proved to be nonmonotonically associated with mean background signal, with elevated variance among both the lowest and the highest regularized scores. (D) Z-normalization dramatically improved count performance at the 80% accuracy threshold, increasing coverage of putative connected pairs from 164 to 3,117. (E) Z-normalization improved consecutive mutual information coverage from 3,261 to 3,527 putative connected pairs. (F) Transfer entropy (*k* = 1) was not notably impacted by Z-normalization, increasing coverage from 2,986 to 3,048 putative pairs.

Although accounting for the tendency of the neurons to participate in many interactions improved detection, pairs with high background signal were more dispersed around the regression line, especially for *count*_*ij*_. In other words, the variance of *residual(X)*_*ij*_ was dependent on *background(X)*_*ij*_, hence displaying heteroskedasticity. This is evident from examining the residuals plotted against ranked *background(X)*_*ij*_ (Figures 4A–C). These differences in variance confound detection since pairs with high background signal are more likely to exceed selection thresholds. We found that heteroskedasticity affected connected as well as unconnected pairs, thus ruling out simple thresholding.

To adjust for heteroskedasticity, we Z-normalized *residual(X)*_*ij*_ by the geometric mean of the pre- and post-synaptic neurons’ standard deviations. To avoid inflating low variances by dividing by small values, normalization was limited to a minimum divisor. We denote the Z-normalized scores by *norm_residual*(*X*)_*ij*_,

$$norm\_residual{\left(X\right)}_{ij}=\frac{residual{\left(X\right)}_{ij}}{\sqrt{\max(\phi_{ij},\phi_{cutoff})}},$$ (20)

where

$$\phi_{ij}=\sigma (residual{\left(X\right)}_{i,1:N-\{j\}})\cdot \sigma (residual{\left(X\right)}_{1:N-\{i\},j}),$$ (21)

and *σ* denotes the standard deviation.

$$\phi_{cutoff}=median(\phi_{ij}).$$

**Figure 5.**
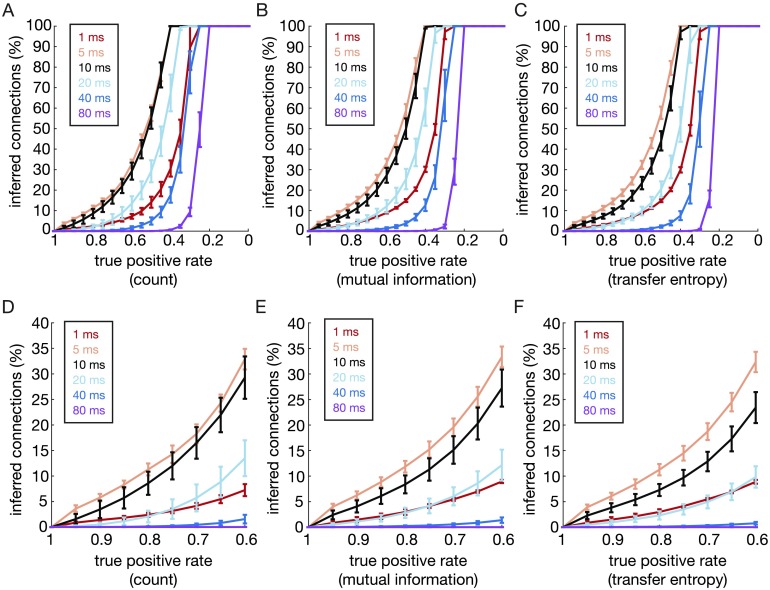
Survival curves for *norm_residual*(*count*)_*ij*_, *norm_residual*(*cMI*)_*ij*_, and *norm_residual*(*TE*1)_*ij*_ for multiple time resolutions and accuracy rates. (A) Count performs best at 5 and 10 ms, revealing almost 50% of connections in the recruitment network. (B) consecutive MI does similarly to count, with 5- and 10-ms time resolution achieving the best performance. (C) Same as in A and B for transfer entropy (*k* = 1). (D–F) Zoom-in of A–C, respectively, showing true positive rates from 0.6 to 1. Note that inference algorithms calculated with 1-ms time-bins display performance in par with 20-ms time-bins. Mean and standard deviation across six simulated datasets are illustrated throughout.

Accounting for scaled variance in background timing relationships, this refinement further improved the coverage of measures (Figures 4D and 4F). The result was particularly dramatic for the count measure. Its success is particularly surprising since *count*_*ij*_ was initially a poor indicator of underlying connection. We suggest that z-scored count estimates provided a simple and powerful first-pass approach to synaptic inference. The performance of this final stage of refinement across timescales and accuracy thresholds is shown in Figures 5A–F). As we previously reported (Chambers & MacLean, 2015), a 1-ms lagged relationship between neurons is less informative compared with inference based on longer time-bins because of the time constant of synaptic integration. Owing to this finding we used time resolutions varying between 5 and 80 ms for all subsequent analyses.

**Figure 6.**
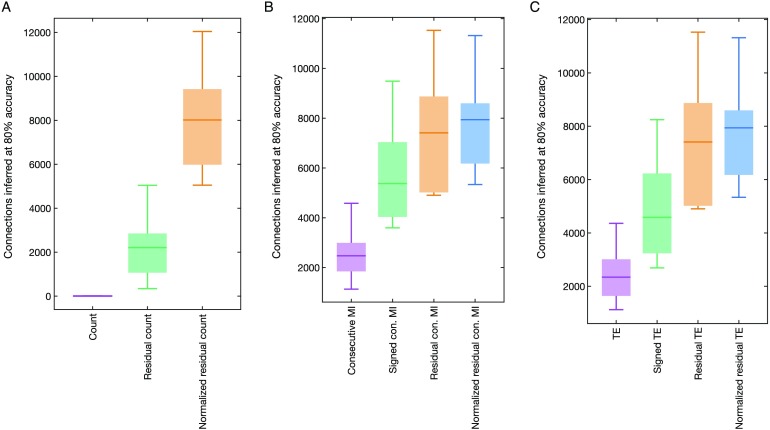
Comparing gains in the regularization pipeline. (A) Inference based on raw count initially achieved zero coverage at the 80% accuracy threshold. Removal of background signal improved coverage substantially, and the greatest gains resulted from Z-normalization to compensate for heteroskedasticity. After regularization, count performed as well as the best other individual inference algorithms. (B) Regularization was also beneficial for the consecutive mutual information measure, with the greatest gains achieved by signing raw scores to distinguish positive timing relationships from negative ones. (C) Transfer entropy (*k* = 1) exhibited similar improvements during regularization, benefiting from signing and removal of background signal.

Average performance gains at 80% accuracy at each stage of refinement collapsed across models for *count*_*ij*_, *cMI*_*ij*_, and *TE*1_*ij*_ are shown in Figure 6. The largest improvement to information theoretic measures resulted from accounting for the interaction sign, whereas coverage for *count*_*ij*_ increased mainly because of Z-normalization of the residuals, bringing *norm_residual*(*count*)_*ij*_ detection in par with *norm_residual*(*cMI*)_*ij*_ and *norm_residual*(*TE*1)_*ij*_. We next investigated whether statistical differences in the collections of predicted synaptic pairs persisted after signing, adjusting for background timing relationships, and selection by thresholding.

### Comparing Similarity and Temporal Preferences Across Measures

**Figure 7.**
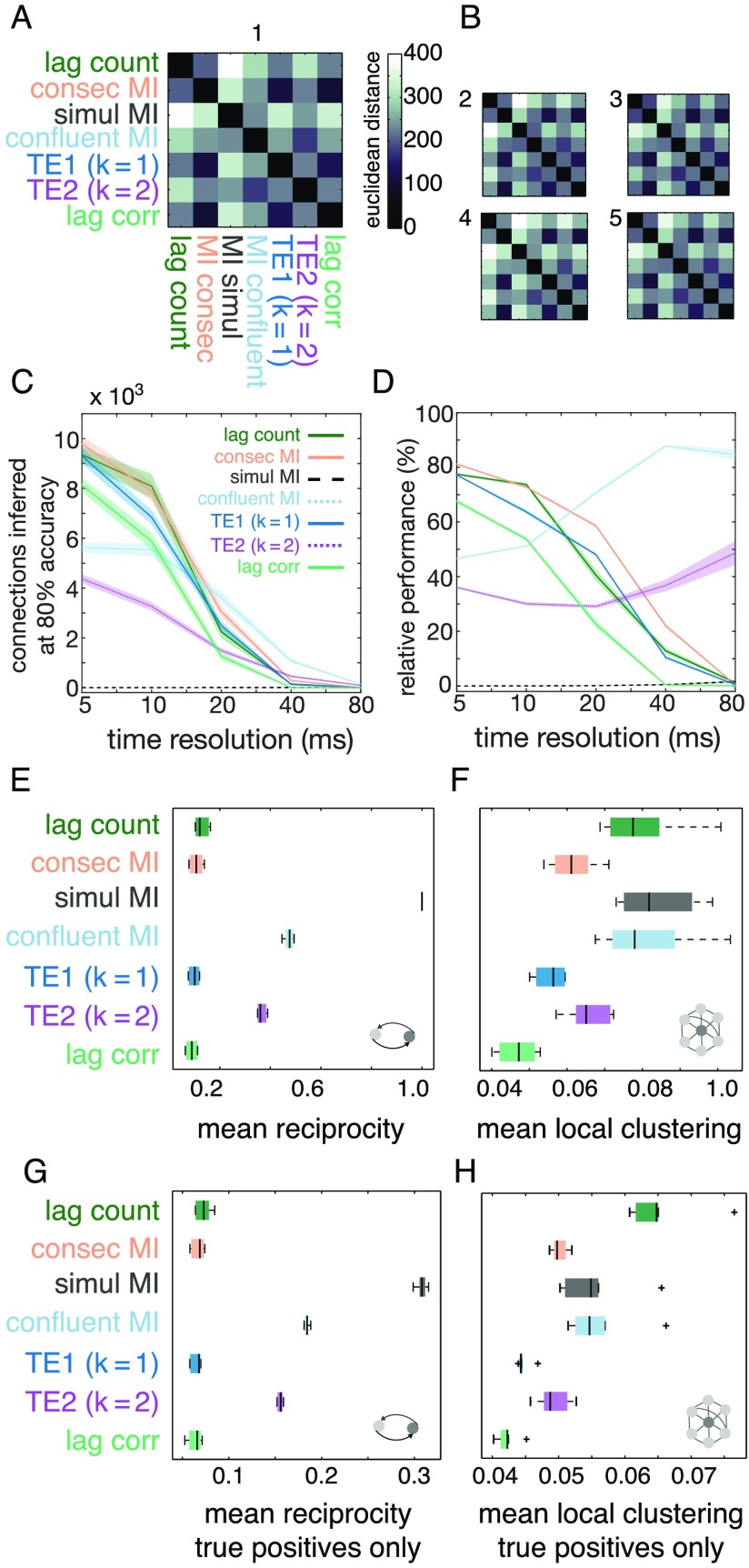
Inference algorithms differ in their statistical preferences. (A) Comparing the strongest putative connections inferred by each regularized inference algorithm, after sparseness-matching and binarization, on the basis of their pairwise Euclidean distances. (B) Stable similarity and dissimilarity relationships manifested across simulated datasets. (C) Inference algorithms exhibited diverse levels of coverage at the 80% accuracy threshold, with all algorithms except simultaneous mutual information performing better at high temporal resolutions. Regularized count, consecutive mutual information, and transfer entropy (*k* = 1) were the best performing measures given high temporal resolution, while confluent mutual information was the best performing measure in conditions of coarser temporal resolution. Lines and shading represent means and standard errors across six simulated datasets. (D) The relative contribution of each inference algorithm to the coverage pooled across all algorithms revealed that measures considering more than one time-bin such as confluent mutual information and transfer entropy (*k* = 2) were able to infer more connections at coarser time resolutions. Lines and shading represent means and standard errors across six models. (E) Different levels of reciprocity were observed across collections of putative connections inferred from different regularized algorithms. Implausibly high reciprocity characterized simultaneous mutual information, because of its emphasis on bidirectional relationships. (F) Levels of mean local clustering differed across regularized algorithms to a lesser degree, with simultaneous mutual information exhibiting highest levels of clustering. (G) Comparison of reciprocity was repeated after excluding false positives and rematching for sparseness. High levels of reciprocity continued to characterize simultaneous mutual information, revealing its strong bias for true reciprocally connected pairs. (H) Comparison of local clustering was repeated after excluding false positives and rematching for sparseness. Elevated local clustering among simultaneous mutual information was revealed to be partially an artifact of its insensitivity to directionality. Collections of true inferred connections were most clustered for the lagged count measure, and least clustered for the lagged correlation measure.

We compared the collection of strongest pairwise relationships for each regularized inference measure. Thresholding was performed independently for each measure to yield sparseness-matched binary subgraphs. We first compared the similarity of detected synaptic connections between each pair of measures. Different measures of pairwise timing statistics highlighted nonidentical, overlapping collections of putative synaptic pairs (Figure 7). Qualitatively, L2 distances between measures were stable across simulated datasets (Figure 7A and 7B). Interestingly, count and simultaneous mutual information (sMI) were most dissimilar, reflecting sensitivity to different temporal structure. Consistent with this interpretation, the most similar measures were correlation, consecutive MI, and TE1.

To investigate further, we compared the performance of each inference metric across timescales. The mean and standard error of coverage and relative coverage are shown in Figures 7C and 7D. An interesting trend was revealed: At high temporal resolution, the measures focusing on consecutive time-bins, namely count and consecutive MI, performed best. But as time resolution decreased, optimal performance shifted increasingly towards measures that combine consecutive and simultaneous time-bins, such as confluent MI and TE2. For small bins, synaptic integration and recruitment often straddle time-bin borders; whereas for large bins, a given synaptic interaction is more likely to occur entirely within single time-bins. In addition to choice of statistical measure, performance depends on the correspondence between relevant timescales of synaptic integration versus the timescale of spike binning (Chambers & MacLean, 2015).

Of all the measures, simultaneous MI was unique in that it contains only information on the spikes that occur in the same time-bin (with no consecutive time-bin information). One might hypothesize that, with large time-bins, sMI would therefore best detect synaptic interactions. However, its inherent symmetry gave rise to frequent errors in directionality. These errors in assuming bidirectional connectivity prevented it from significantly crossing the 80% accuracy threshold, and it thus achieved zero coverage at this cutoff. While sMI is fully symmetric in its raw form, the normalization process removed this symmetry, which explains how the refined score achieved nonzero coverage at the largest timescale.

### Comparing Topological Preferences Across Measures

We hypothesized that the inferred subgraphs of synaptic connections might differ in systematic ways depending on the algorithm employed and that these differences would be detectable by comparing the topological organization of the subgraphs. If inferred networks differed in the lagged relationships that they were sensitive to, it was possible they contained complementary information about the location of true connections. In previous work we had found that specific topological motifs found in simulated model activity were also found in experimental data collected from somatosensory cortex, demonstrating that higher order dynamical organization has the potential to generalize across network construction (Chambers & MacLean, 2016). We compared estimates of reciprocal connection probability and local clustering to understand whether different measures made similar estimates of these quantities (Methods). Inferred topologies were characterized by a diversity of reciprocity and local clustering (Figure 7E–H). Unsurprisingly, the *simultaneous MI* measure exceeded all other measures in the level of reciprocity represented among its strong entries, since through symmetry it tends to predict bidirectional connectivity. Note that ground-truth reciprocity is 0.2. In contrast, measures sensitive to time-lagged statistical relationships tended to be characterized by lower levels of reciprocity (Figures 7E and 7G). Since pairwise reciprocity sets a lower bound expectation for local clustering, it is not surprising that measures followed a similar rank ordering for both estimates, although fractional differences were smaller for estimates of local clustering. Emphasizing that the two metrics are related but distinct, we note that count estimates of local clustering were relatively high in relation to its estimate of reciprocity. Overall, inferred topologies exhibited nonidentical statistical features depending on the algorithm employed.

We next tested whether these differences were dominated by detection errors, while encompassing statistically similar subsets of true synaptic connections. To investigate, we repeated the analysis above for true positives only. After this step, inferred topologies were matched in sparseness by thresholding and binarized to prevent any uncontrolled differences in edge density. These two steps, thresholding and binarization, were conducted solely for these comparisons (Methods). After pruning false positives from inferred topologies, estimates of reciprocity obeyed a qualitatively similar rank ordering (Figure 7G); sMI continued to be dominated by reciprocal edges even after pruning false positives, exhibiting a strong selective preference for bidirectional synaptic pairs. Although it does not faithfully represent the ground-truth statistics of synaptic recruitment (investigated below), this feature could be useful in applied experimental settings, for example, for targeting multicellular patch clamp recordings to find reciprocal pairs, potentially generating higher experimental yields. In contrast, after pruning false positives, sMI exhibited far lower local clustering (Figure 7H), revealing that false inference of reciprocal connectivity made a misleading impact in quantifying local clustering. The count subnetwork identified connections related by high local clustering. Correlation and TE1 measures identified synaptic pairs less likely to be bidirectionally connected or clustered tightly together. Since different inference measures appeared to prefer distinct subsets of the synaptic network, we next tested whether their heterogeneous strengths could potentially be pooled to yield higher sensitivity than was achievable with any measure alone.

### Ensemble Approach for Combining Measures

We found that each inference algorithm revealed overlapping but distinct sets of causal connections with different biases. This diversity suggested that an ensemble approach, leveraging complementary sources of information across measures, could potentially improve upon inferences based on any single measure. We employed a stochastic search strategy (Methods) to find a weighting scheme for combining measures. The weights we obtained largely paralleled the independent performance of the component measures (Table 4), with consecutive binning being favored at high temporal resolution and confluent or simultaneous binning being favored at low temporal resolutions. Surprisingly, significant weight was assigned to sMI at larger exposures despite sMI not being able to achieve any real coverage on its own (see Figures 7C and 7D). This result emphasized the utility of ensemble approaches in cases in which low-performing algorithms can still improve the ensemble performance.

**Table 4.** Ensemble weights

	5 ms	10 ms	20 ms	40 ms	80 ms
Count	0.1411 ± 0.084	0.1990 ± 0.125	−0.0029 ± 0.116	0.0025 ± 0.051	0.0199 ± 0.037
Consecutive MI	0.2782 ± 0.094	0.2396 ± 0.116	0.2098 ± 0.071	0.1335 ± 0.129	0.1260 ± 0.121
Simultaneous MI	0.0035 ± 0.016	0.0080 ± 0.015	0.0591 ± 0.048	0.2496 ± 0.125	0.2607 ± 0.112
Confluent MI	0.2058 ± 0.064	0.2260 ± 0.080	0.2487 ± 0.111	0.1841 ± 0.120	0.2696 ± 0.117
TE (*k* = 1)	0.1084 ± 0.118	0.1116 ± 0.124	0.1484 ± 0.063	0.0884 ± 0.062	0.0323 ± 0.057
TE (*k* = 2)	−0.1086 ± 0.036	0.0359 ± 0.066	0.0984 ± 0.082	0.1446 ± 0.081	0.1191 ± 0.092
Correlation	−0.1302 ± 0.046	−0.1052 ± 0.092	−0.1329 ± 0.083	−0.0555 ± 0.128	−0.0527 ± 0.108

Data reported here as mean ± *SD* across six simulated datasets; each ran five times through simulated annealing.

**Figure 8.**
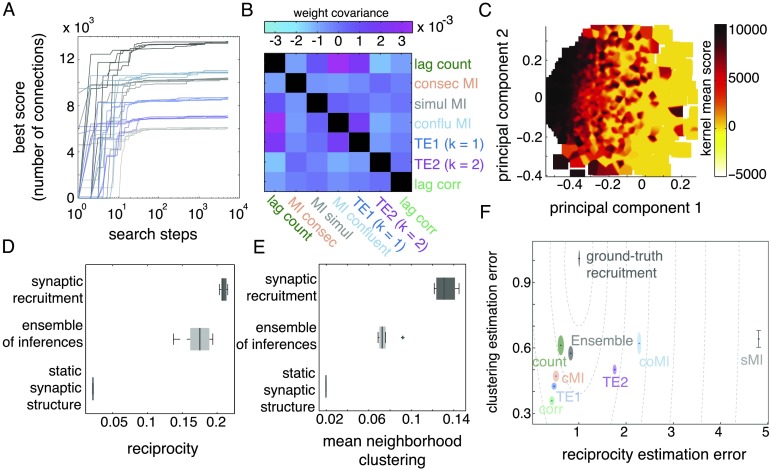
Pooling individual measures to generate ensemble predictions. (A) Optimal weightings were obtained using random-walk search with simulated annealing on step sizes. Independent searches converged on similar best scores for each simulated dataset, grouped by color. Appropriate weights were discovered early, but modest gains persisted throughout the search procedure. (B) Covariance matrix quantifying common structure in weights throughout the search procedure, after concatenating all simulated networks and trials. Entries along the main diagonal were masked out for visualization purposes. (C) Among pooled weights, good scores tended to cluster along one extreme of the first principle dimension, while robust to differences along the second principle dimension. Good solutions also appeared in isolated peaks throughout the solution space. Diverse weighted combinations yielded good performance in inferring synaptic connectivity. (D) Ensemble-generated topologies closely reflected the ground-truth reciprocity statistics of synaptic recruitment in the simulated network. Synaptic recruitment preferentially occurred within a nonrepresentative subset of the underlying random structural network. (E) Like the ground-truth network of synaptic recruitment, ensemble-generated topologies were characterized by elevated local clustering. However, like the best individual measures, ensemble scores somewhat underestimated its true extent. (F) With respect to reciprocity and local clustering, the two best characterizations of synaptic recruitment statistics were achieved by count and ensemble inference.

Weights were pooled over all models for principal components analysis (PCA), to identify model-independent features of the score landscape. Weight covariance for 10-ms simulated temporal resolution is shown in Figure 8B, with entries in the main diagonal masked out for visualization purposes. The measures count, confluent MI, and TE1 exhibited particularly high covariance, suggesting that they provide complementary information about synaptic connections. This observation is consistent with their different statistical preferences. TE2 tended to covary negatively with these measures, suggesting it was being leveraged to disambiguate pairs without a true connection. The complementary information yielded by TE1 versus TE2 reiterated the power of studying functional coupling at multiple time-lags (Ito et al., 2011). The search over weight space is illustrated for the first two PCA dimensions with performance indicated by color (Figure 8C). Although score was not factored explicitly into the dimensionality reduction, effective weightings clustered together as a function of the first principle dimension, suggesting a large basin of feasible weights. Diverse combinations of weights had the potential to pool measures productively.

### Improved Sensitivity With and Generalization of Ensemble Inference

**Figure 9.**
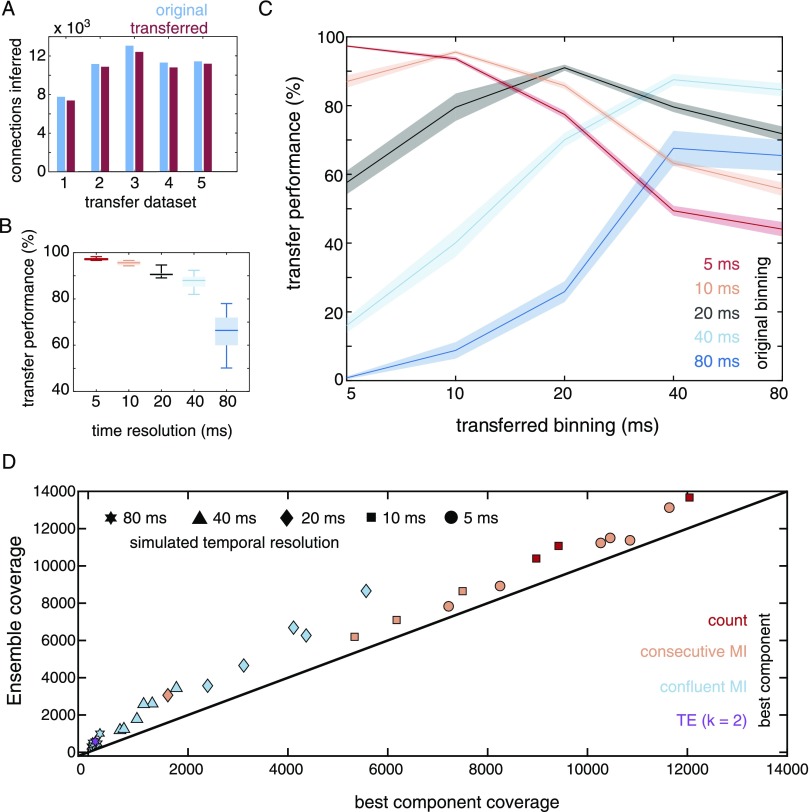
Ensemble weightings generalize across simulated datasets and outperform individual measures. (A) Ensemble weightings were optimized for number of connections inferred using stochastic search (original performance, blue). Weights found for a random dataset with 5-ms time resolution were used to construct ensemble-generated adjacency matrices for each other dataset also binned at 5 ms (transfer performance, red). Transfer performance approached the original performance based on optimized weighting schemes. (B) Given matched temporal resolution, transfer performance was nearly as good as original performance for fast temporal resolution. At slower time-bins, transfer performance was somewhat less effective. (C) Transfer performance was best when temporal resolution was matched between original and transferred recordings, and mismatch degradation was worst for weights originally learned from recordings with slow temporal resolution. Line and shading represent means and standard errors across six simulated datasets. (D) Ensemble-generated topologies outperformed their best component measures across all bin sizes and model repetitions. Component measures with the best individual performance varied, but regularized count, consecutive mutual information, and confluent mutual information were often the best performing individual measures. Algorithms incorporating information at multiple timescales fared well, including confluent mutual information and transfer entropy (*k* = 2).

As we have previously reported, the recruitment network is characterized by elevated clustering in the local synaptic neighborhood (Chambers & MacLean, 2016). The ensemble method recapitulates these features better than the best individual measures (Figures 8D–F). The ensemble also exhibited improved sensitivity at the 80% precision cutoff, with larger relative improvements as sampling rates decreased. To examine the improvements offered by the ensemble method, we plotted the coverage for the ensemble score compared with its best performing component score (Figure 9D). Across all simulated networks, all trials, and all exposures, the ensemble method increased coverage, with absolute gains being around 1,000 neuronal pairs over the best measure. Given the low component performance at large exposures, this represented a larger relative gain at these timescales, which is relevant for inferring connections using common experimental imaging techniques.

To test whether weights learned for one simulated dataset could transfer to other simulated networks, we computed the ensemble scores for each using weights learned for different datasets (transferred weights) and compared the performance to the datasets’ performance with their own weights (original weights). Simulated temporal resolution was matched for the transferred and original sets of weights. We found weights trained on one simulated network approached coverage after transfer to another network, with 97.32 ± 0.54% (mean ± *SD*) of pairs inferred with original weights also inferred with transferred weights, at 5-ms time resolution (Figures 9A and 9B). Generalization of weights across datasets depended on timescales in a similar manner to overall performance. Nonetheless, retained coverage was still impressive at 40 ms, with over 85% of inferred connections preserved (Figure 8B), suggesting that simulations of realistic networks may be exploited to train ensemble weights for experimental data, even in cases in which temporal resolution is limited.

We also explored whether weights can be transferred across both simulated network and timescales by examining retained performance after transfer from nonmatching time resolution. Once again, the ensemble method was robust to weights generalization, with transferred performance remaining above 80% for timescales that are similar yet not identical to the timescale the measures were originally computed with (Figure 9C). For example, models binned at 10 ms with weights transferred from 5- and 20-ms models performed at 87.002 ± 3.578% and 86.744 ± 1.890% of their original performance, respectively. Ensemble weights generalized across simulated datasets, and matching temporal resolution at least coarsely, was advantageous for transfer performance.

## DISCUSSION

Within local cortical circuits, spiking activity propagates through synaptic networks in order to implement computation and shape behavior. Yet individual connections are weak in isolation, and patterns of coordination are complex and variable. Activity, or functional, mapping approaches, such as those presented here, infer probable synaptic recruitment patterns from statistical regularities in spike timing. In this framework, statistical relationships are leveraged to predict synaptic connections, typically by thresholding to isolate the most reliably coupled pairs. Importantly, functional graphs, which succinctly summarize circuit dynamics, identify the synapses that are actively involved in the recruitment of post-synaptic neurons; that is, those synapses that drive the post-synaptic neuron to threshold. Explicitly, only those connections revealed by (a) the timing of pre-synaptic action potentials, (b) the integrative properties of the post-synaptic neuron, and (c) the membrane potential of the post-synaptic neuron can ever be recovered by an inference approach. These are a limiting set of criteria that dramatically lessen the connections that can be recovered using these methods. Consequently, these approaches are not a realistic means to fully reconstruct a synaptic wiring diagram. Rather, this is an approach that identifies synapses involved in implementing computation, and transmitting information during the specific epoch of dynamics that the graph summarizes, that is, the recruiting network. As a result, these connections are particularly interesting from a functional perspective. In this work, we compared methods for inferring excitatory synaptic connections, in order to understand the strengths and weaknesses of each. For mapping activity propagation through networks, we present two approaches to improve the inference of excitatory connections: a regularization pipeline to improve the performance of individual inference algorithms, and an ensemble stacking procedure that combines the best features of diverse measures.

### Refinements of Inference Measures

To improve inference of excitatory connections, we applied a sign to the information theoretic measures which disambiguates positive interactions from negative interactions. We note that negatively signed interactions could potentially provide insight into inhibition within the network, a long-standing challenge in connectivity research, but also point out that disambiguating active inhibition from lack of excitation is a nontrivial endeavor. In this work we focus on the more tractable goal of mapping excitatory connections alone. Assigning valence to inferred relationships enhanced our recovery of excitatory connections.

Inference algorithms were further strengthened by removing a source of noise, reflecting background timing coincidences, not reflecting monosynaptic interactions. After reexpressing measures to conform better to normality, we regressed out the mean component of this background signal by averaging over pre- and post-synaptic weighted degree. Since connectivity is sparse even among near neighbors, this averaging procedure was dominated by background influences specific to each neuron’s firing rate and response profile. This tendency was not linear in magnitude across degrees, however, resulting in heteroskedasticity of residual scores. We corrected for the variability in standard deviation over the residuals with Z-normalization, which further increased the accuracy of inferred interactions. This step was particularly effective for count, transformed by regularization into one of the single most effective indicators of connectivity. This approach is similar in spirit to the normalized count procedure described in prior work (Pajevic & Plenz, 2009). These steps can be thought of in terms of informing inference algorithms not only by regularities within specific connections, but also by the statistics of the entire network. In this regard the current study differs from previous inference attempts in neuronal networks and, particularly, improves on previous procedures for thresholding. We suggest similarly inspired next steps would be to incorporate priors about higher order structures such as motifs and clusters as well as accounting for lognormal distribution of weights (Song et al., 2005) to further facilitate detection.

### Ensemble Method

Different inference algorithms capture and summarize subtly different attributes of collective activity, and it is important to be mindful about these features when interpreting functional connectivity. Temporal resolution appears to be a particularly key design variable, and bin sizes of 25–50 ms or smaller are preferable for identifying synaptic connections, likely reflecting synaptic integration times (Chambers & MacLean, 2015). Of course, even at much slower temporal resolution, the same algorithms can be useful for quantifying average timing relationships among active neurons. For mapping population dynamics, our results suggest that a host of productive statistical measures exist, which can be leveraged to infer likely patterns of synaptic recruitment. These measures are useful in isolation, and they become even more incisive in combination.

We found that each inference method isolated collections of putative underlying synaptic connections that are nonidentical. This is an ideal situation for the application of ensemble methods. Here we used linear combinations of multiple measures in order to improve predictions of putative synaptic connections beyond the best single inference method. Nonlinear combinations of measures have the potential to synergize further, leading to further gains in performance, but will face increasingly severe difficulties in generalization—a problem typical of classification in high-dimensional spaces given limited training data (Vapnik, 2013). Ensemble approaches employ a diversity of methods for pooling, and one common method is majority vote on individual classifiers (Liaw & Wiener, 2002). However, ensemble approaches are also frequently applied to real-valued outputs rather than binary classifier decisions (Mendes-Moreira, Soares, Jorge, & De Sousa, 2012). One of the most common ways to leverage multiple classifiers in combination is known as bagging, where the ensemble score is the mean over all real-valued scores of individual measures. We demonstrate that better performance can be obtained in a weighted combination of the inference approaches. This latter framework for ensemble learning is known as stacking. In general, stacking algorithms are characterized by a pooling step, known as a combiner algorithm. As an example, high performers in the Netflix Prize employed linear regression and, later, backpropagation in a feedforward neural network for their combiner algorithm (Töscher, Jahrer, & Bell, 2009). In order to optimize stacking weights, we employed an annealing random walk search strategy, a common approach in the field of ensemble learning, including evolutionary approaches such as genetic algorithms and stochastic hill climbing (Ruta & Gabrys, 2005). The question of how best to pool diverse connectivity inference algorithms remains an area necessitating further study. In practice, since recording conditions vary from one lab to another, experimenters with different experimental protocols than the ones studied here *in silica* should retrain a combiner algorithm based on their own internal experimental design and selection of available inference algorithms with the understanding that each algorithm has different biases and the appropriate ensemble will depend on the expected underlying connectivity and time constant of synaptic integration.

The measures we studied in this work framed inference of synaptic connectivity as a binary classification algorithm. However, inference has the potential to also be cast as a regression problem using some continuous-valued measure of pre-synaptic influence (e.g., induced excitatory post-synaptic potential), which may lead to further gains, particularly in coverage. However, scarcity of ground-truth data and population-level coordination (obstacles facing any connection-inference approach) will make it challenging to move beyond binary classification to infer true synaptic connections. In addition to the algorithms we compared in this work, a host of other effective approaches have been described in the literature, including particle methods (Gerstein & Aertsen, 1985), generalized linear models (GLMs; Gerhard et al., 2013; Zaytsev, Morrison, & Deger, 2015), and Bayesian methods (Chambers & MacLean, 2015; Mishchencko, Vogelstein, & Paninski, 2011; Pajevic & Plenz, 2009). It may be that ensemble predictions about underlying connectivity could be improved further by including these approaches in the pooling step.

### Inference for Circuit Reconstruction/Insights Into Information Processing

Understanding how connection structure gives rise to synaptic recruitment remains a central goal for the study of neocortical circuits. Computation and behavior are enacted by propagating activity, so understanding synaptic recruitment mechanistically within active cortical networks is fundamental to the study of behaving animals. The importance of this issue was recognized early (Gerstein & Perkel, 1969; Palm, 1982), but technical obstacles limited its active study. In the last five years, progress is being made in this area through the study of functional relationships in active populations. In other words, it is important to consider not only whether a connection is present, but also which connections are coactive or otherwise functionally related, and causal to spike propagation in a given context.

A confluence of evidence argues that the function of an individual connection depends on its arrangement within the local synaptic neighborhood. For example, a given connection will make a different impact if it is isolated versus arranged within a local cluster (Pajevic & Plenz, 2012). In neural cultures, frequent ignition sites were associated with elevated local clustering, for which a model of convergent amplification was proposed (Orlandi, Soriano, Alvarez-Lacalle, Teller, & Casademunt, 2013). Culture activity maps were characterized by elevated clustering and short mean path lengths (Pajevic & Plenz, 2009). Intriguingly, clustering motifs, reciprocity, and heavy-tailed weight distributions may emerge through self-organizing plasticity processes (Miner & Triesch, 2016). These nonrandom features have received high levels of interest and may be consistent with several global topological organization schemas (Vegue et al., 2017). In neocortical tissue from mouse sensory cortices, spontaneous lagged firing relationships were found to be characterized by elevated modularity and hierarchical features (Gururangan, Sadovsky, & MacLean, 2014; Sadovsky & MacLean, 2013). Similarly, multielectrode array recordings were marked by rich club structure (Nigam et al., 2016) and broad degree distributions (Timme et al., 2016). In the latter work, functional hub neurons played a crucial role in supplying inputs to computationally important neurons downstream. Related theoretical work suggests functional hubs may take on distinct roles shaped by their assortativity or disassortativity relationships (Piraveenan, Prokopenko, & Zomaya, 2012). Converging sources of evidence have identified generalizable nonrandom features within connected neural systems consistent with the idea that these are key features to consider when describing the flow of activity through the circuit. Beyond these higher order functional relationships, we have previously shown that inference is biased towards stronger connections (Chambers & MacLean, 2015). As a result, inference methods sometimes pose difficulties in interpretation (James, Barnett, & Crutchfield, 2016), and they are limited in accuracy as well as sensitivity to weak synaptic connections, which are crucial for realistic spiking dynamics (Sadovsky & MacLean, 2013; Teramae, Tsubo, & Fukai, 2012). Continued development of tools to understand the large-scale organization of synaptic networks is an important area for further investigation.

The function of individual connections also depends on recent dynamics of the local circuit. On short timescales, unexpectedly effective recruitment can arise when inputs to a neuron are temporally coordinated (Rossant, Leijon, Magnusson, & Brette, 2011), for example, through the channel dynamics of action-potential generation (Fontaine, Peña, & Brette, 2014) or through interactions with the dendritic arbor (Major, Larkum, & Schiller, 2013). Higher order connectivity, particularly fan-in clustering, may favor coordinated inputs in this way, supporting effective synaptic integration (Chambers & MacLean, 2016). On moderate timescales, short-term plasticity imposes additional complexity in predicting the function of individual connections, where synaptic efficacy depends in part on a hidden state (Buonomano & Maass, 2009). In addition, diverse sources of neuromodulation can reorganize synaptic efficacy in real time, in ways that are difficult to understand a priori. For example, circuit-level reorganization can occur in response to acetylcholine, impacting the salience of extrinsic inputs versus local recurrent drive (Runfeldt, Sadovsky, & MacLean, 2014).

The complexity of structure-function relationships in neocortex is daunting. Isolated structural features can take on unexpected functional roles in the context of the local active network. Yet out of the extreme diversity of mechanisms shaping cellular communication, stable statistical relationships in spike timing emerge. Maps of synaptic recruitment are a promising avenue to summarize the complex effects imparted by these many interlocking subcellular processes. For progress to continue in understanding large active neural circuits, it is important that statistics of spike timing among large populations continue to be investigated actively by the neuroscience community.

## AUTHOR CONTRIBUTIONS

Brendan Chambers and Maayan Levy contributed equally. Brendan Chambers: Conceptualization; Data curation; Formal analysis; Investigation; Methodology; Software; Validation; Visualization; Writing – original draft; Writing – review & editing. Maayan Levy: Conceptual ization; Data curation; Formal analysis; Investigation; Methodology; Software; Validation; Visualization; Writing – original draft; Writing – review & editing. Joseph B. Dechery: Con ceptualization; Validation; Writing – original draft; Writing – review & editing. Jason N. MacLean: Conceptualization; Data curation; Funding acquisition; Investigation; Methodology; Project administration; Resources; Software; Supervision; Validation; Writing – original draft; Writing – review & editing.

## TECHNICAL TERMS

Recruitment network: Contains only synapses that directly contribute to post-synaptic firing since these are the only synapses that can be inferred.
Inference methods/algorithms/measures: Capture statistical dependency between the spike trains of two neurons. For example, mutual information and correlation.
Lagged firing: Two neurons spiking in consecutive time-bins.
Ensemble method/approach: A machine-learning-inspired approach in which multiple inference methods are pooled.
Motif: A pair or triplet of neurons connected in a certain pattern. For example, a fan-in triangle.
Stacking: Generating one score from multiple inference methods by calculating a weighted sum.
